# Determinants of change in unintended pregnancy in Ethiopia using the 2005 and 2016 EDHS: non-linear multivariable decomposition analysis

**DOI:** 10.1186/s13690-022-00984-2

**Published:** 2022-11-10

**Authors:** Abiyu Abadi Tareke, Ermias Bekele Enyew, Abiy Tasew Dubale, Aynadis Worku Shimie, Mulugeta Desalegn Kasaye, Habitu Birhan Eshetu

**Affiliations:** 1 Department of Health Management Information System, West Gondar Zone Health Department, Gondar, Ethiopia; 2 Department of Health Informatics, Mettu University, Mettu, Ethiopia; 3 Department Health Informatics, Medicine and Health Science College, Debre Markos University, Debre Markos, Ethiopia; 4 Department of Health Informatics, Wollo University, Dessie, Ethiopia; 5 Department of Health Education and Behavioral Sciences, Institute of Public Health, College of Medicine and Health Sciences, University of Gondar, Gondar, Ethiopia

**Keywords:** Unintended pregnancy, Multivariable decomposition, EDHS, Ethiopia

## Abstract

**Background:**

Unintended pregnancy is a pregnancy either mistimed or unwanted. The main consequence of unintended pregnancy is inducing abortion. In Ethiopia, more than half of unintended pregnancies end up in abortion.

**Objective:**

This study aims to measure the change in unintended pregnancy among women of reproductive age between survey years 2005 and 2016 and to identify the socio-demographic factors that most significantly contributed to the change.

**Methods:**

Data from the two most recent Ethiopian Demographic and Health Surveys (EDHS) were analyzed. We quantified the contribution of socio-demographic factors in the change of unintended pregnancy, using Oaxaca-Blinder decomposition for non-linear regression models by applying the STATA command ‘mvdcmp’.

**Result:**

Unintended pregnancy decreased from 37% in 2005 to 27% in 2016 in Ethiopia. Both changes in population characteristics and coefficient were the contributing elements to the observed change in unintended pregnancy. Among population characteristics factors, being a partial decision-maker and being a slum in the Somali region contributed 10 and 14% to the change of unintended pregnancy between the 2005 and 2016. Of the coefficient factors, knowledge of modern family planning, being a partial decision-maker, media exposure, distance to health facilities, and health facility visits contributed to the change by 93, 43, 17, and 10% respectively.

**Conclusion:**

The majority of the change in unintended pregnancy from 2005 to 2016 survey was due to differences in coefficients (85%). The principal contributing factors to the change of unintended pregnancy were FP knowledge, decision making, media exposure and health facility visits. Therefore, an interventional plan will be efficient, better, and more effective if focused on the larger contributing factors.

## Introduction

As defined by the Centers for Disease Control and Prevention (CDC), unintended pregnancy is defined as a pregnancy that is either unwanted, or the pregnancy is mistimed, such as the pregnancy occurring earlier than desired [1].

Worldwide, the rate of pregnancy shows significant decrement, but the rate of unintended pregnancy remains high. From 1990 to 2015 the rate of unintended pregnancy declined 15 percentage points (i.e. from 79 to 64%) [2]. Globally, of the 208 million pregnancies that happened in 2008, 86 million (41%) were unintended and 40% of the total pregnancies in developing countries were unintended [3]. Similarly, Of these 85 million unintended pregnancies that occurred in 2012, 50% they resulted in abortion, 13% ended in miscarriage, and 38% resulted in an unplanned birth [4]. Nearly 40% of unintended pregnancies ended in abortion from 2015 to 2019 [2]. The burden of unintended pregnancy remains high in Africa. According to a study done using multiple sources of data to estimate unintended pregnancy, 35% of the total pregnancies in Africa were unintended.

Pocket Studies done in different portions of Ethiopia showed a high prevalence of unintended pregnancy. For instance, a study conducted in the central portion of the country revealed that the prevalence of unintended pregnancy was 41.5% [5]. Other studies also reached a similar conclusion were; Gelan District (55%) [6] Hadiya zone(36%) [7], Gondar city (47%) [8], and Harari (33%) [9]. The consequences of unintended pregnancy are eclampsia, preeclampsia, and hemorrhage after birth, relative to planned pregnancy [10]. But, the most unpleasant outcome of unintended pregnancy is unsafe abortion [11, 12]. In Ethiopia, more than 50% of unintended pregnancies end up in induced abortion [9].

Much kind research revealed that socio-demographic factors such as; maternal age [5, 13, 14], level of education [13, 15–17], religion [18–20], marital status [5, 21, 22], distance from the nearest health facilities, parity [5, 13, 16, 18], household size [22, 23], wealth status [20], knowledge on ovulation cycle, knowledge on family planning [16], ever had terminated pregnancy [5, 14, 24] residence [16, 18, 20] and region [13, 23] were the main factors associated with unintended pregnancy. Therefore, to close the huge gap in this country, major contributing factors to the decrease or increase of unintended pregnancy need to be identified using nonlinear regression methods.

There hasn’t been any study that used broadly representative data to identify the contributing factors to the Change in unintended pregnancy. In Ethiopia, prior studies were mainly focused on the prevalence and determinant factors of unintended pregnancy using a dataset of a single survey. The factors which contributed to the change of unintended pregnancy remained untouched. Therefore, this study is aimed to address the contributing factors for the changes in unintended pregnancy through multivariable decomposition analysis based on the 2005 and 2016 EDHS.

This study applied multivariable decomposition analysis to identify which socio-demographic predictors are strongly correlated with the change in unintended pregnancy among reproductive-age women (15–49 years) in Ethiopia. We hypothesized that the change of unintended pregnancy changed in Ethiopia between the two survey periods and different factors contributed to the change of unintended pregnancy.

## Method and materials

### Study design and sampling procedures

The data utilized for this study were retrieved from 2005 and 2016 EDHS. These surveys were carried out using a stratified two-stage random sampling method. In the first stage, 540 Enumeration Areas (EAs) in EDHS 2005 and 645 EAs in EDHS 2016 were randomly selected proportional to their EA size and, on average, 27 to 32 households per EAs were selected in the second stage.

A weighted sample of 14,890 (7300 in survey 2005 and 7590 in survey 2016) reproductive-age women were incorporated in this study. Sample weighting was applied to correct for the under or over-sampling of different strata during sampling. If weights had been used, all calculations would have been biased towards the levels and relationships in the over-sampled strata. Therefore, weighting the sample helps in restoring the representativeness of the survey and in getting reliable statistical estimates. Detailed information about sampling procedures was showcased in the EDHS reports [25, 26].

### Study variables outcome variable

The outcome variable was unintended pregnancy, which is defined as either unwanted or mistimed pregnancy. Unwanted pregnancy is a pregnancy that took place when no longer child is required by the woman. Mistimed pregnancy is a pregnancy that came earlier than expected time [1]. The outcome variable was dichotomized as “unintended” if women had encountered either unwanted or mistimed last pregnancy were coded 1 otherwise “wanted” code 0.

### Independent variables

The independent variables regarded in this study were: age, educational status, religion, marital status, place of residence, women working status, household wealth status, media exposure, ever termination of pregnancy, knowledge of family planning, history of visiting health facilities in the last 12 months, history of a visit by field workers in the last 12 months, perceived distance from home to proximate health facilities, age at first marriage, parity, sex of household head, region of residency, and desired number of children.

### Operational definition

#### Media exposure

Created by combining whether a respondent reads the newspaper, listens to the radio, or watches television. If the woman was exposed to at least one of the three Media she was considered as “exposed” and otherwise “not exposed” coded “0”. This is done to overcome the reduction of sample size when the three media exposure is used as independent variable separately. For example, only educated women are eligible for the question “do you read a newspaper?” and this will exclude uneducated women. The same is true for watching television, that only women who have access to electricity are suited for this question.

### Statistical analysis

Important variables were extracted from the Individual Record (IR) datasets. Data were weighted using the “svyset” STATA command, and it was applied for descriptive analysis. The variables required for the “svyset” are the weight variable (v005), primary sampling unit (v021), and strata (v023).

### Non-linear multivariable decomposition analysis

Logit-based multivariable decomposition analysis for a non-linear response model was calibrated to analyze the change in unwanted pregnancy between the two surveys. This model was applied to identify the source of variations of unintended pregnancy between the two surveys (i.e.2005 and 2016). The Logit-based multivariable decomposition analysis utilizes the output from the logistic regression model to assign the observed change in unintended pregnancy over time into different components.

Using the Stata command “append,” the 2016 EDHS dataset was appended to the 2005 EDHS dataset for analysis. Moreover, the Logit-based multivariable decomposition analysis was calibrated using the “mvdcmp” STATA command. The change in unintended pregnancy can be explained by the compositional difference (i.e. the difference in characteristics) and/or the difference in effects of explanatory variables (i.e. the difference in the coefficients) between the surveys. For logistic regression, the Logit or log-odd of unintended pregnancy is taken as:

$$\textrm{Logit}\ (2005)-\textrm{Logit}\ (2016)=\textrm{F}\ \left(\textrm{X}2005\upbeta 2005\right)-\left(\textrm{F}\ \textrm{X}2016\upbeta 2016\right)$$

[27].

X indicates independent variables (unintended pregnancy).

β denotes that, the regression coefficient of each selected explanatory variables.

The E component refers to the part of the differential owing to differences in endowments or characteristics. The C component refers to that part of the differential attributable to differences in coefficients or effects.

### Ethical approval and consent

Since the study was a secondary data analysis of publicly available survey data from the MEASURE DHS program, ethical approval and participant consent were not necessary for such kind of study. We requested DHS Program, and authorization was awarded to download and use the data for advance analysis from the website http://www.dhsprogram.com.

## Result

### Characteristics of the study population

Nearly half of the study participants in 2005 (46.4%) and 2016 (50.4%) were aged between 25 and 34 years. The percent of none educated women decreased by 15% from 2005 to 2016 (Table 1).

**Table 1:** Percent distribution of socio-demographic characteristics among respondents, 2005 and 2016 Ethiopian demographic health survey

Variable	2005 EDHS *N* = 7300	2016 EDHS *N* = 7590
**Unintended pregnancy**	37.00%	27.00%
**Maternal age**		
15–24	26.18%	23.77%
25–34	46.38%	50.42%
35–49	27.44%	25.82%
**Educational status**		
None	78.49%	63.12%
Primary	16.48%	28.32%
Secondary & above	5.03%	8.55%
**Religion**		
Traditional	1.55%	1.27%
Orthodox	44.69%	37.97%
Catholic	1.04%	0.94%
Protestant	19.20%	21.76%
Muslim	32.56%	37.21%
**Region of residency**		
Tigray	8.23%	7.62%
Afar	1.15%	1.03%
Amhara	31.81%	26.11%
Oromia	39.04%	40.31%
Somali	4.91%	4.16%
Benishangul –Gumuz	0.95%	1.02%
SNNP	11.11%	16.53%
Gambela	0.14%	0.15%
Harari	0.25%	0.13%
Addis Ababa	1.98%	1.49%
Dire Dawa	0.41%	0.25%
**Wealth status**		
Poor	42.07%	43.55%
Middle	21.73%	20.93%
Rich	36.20%	35.52%
**Place of residency**		
Urban	8.67%	12.77%
Rural	91.33%	87.23%
**Working status**		
Employed	30.93%	46.27%
Not employed	68.96%	53.73%
**Current marital status**		
Married	91.55%	92.49%
Not married	8.45%	7.51%
**Media exposure**		
Not Exposed	62.49%	65.47%
Exposed	37.51%	34.53%
**Autonomy of decision making**		
Not autonomous	35.72%	19.75%
Partially autonomous	50.58%	66.4%
Fully autonomous	13.48%	13.85%
**Desired number of children**		
Above 5+	55.47%	53.80%
Less than 5	44.53%	46.20%
**Health facilities visit in the last 12 months**		
No	69.85%	48.59%
Yes	30.15%	51.41%
**Knowledge of family planning methods**		
No	11.14%	1.33%
Yes	88.86%	98.67%
**Perceived distance to proximate health facilities**		
Big problem	73.24%	58.06%
Not big problem	26.76%	41.94%

### Change of unintended pregnancy

The magnitude of unintended pregnancy decreased from 37% in 2005 to 27% in 2016 surveys (Table 1).

## Decomposition analysis

Variables with a *p*-value less than 0.2 from the bi-variable decomposition analysis were selected as candidate variable for multivariable decomposition analysis. Because there is a greater amount of missing value, the variable parity is dropped entirely. Additionally, the variable region, religion, and place of residence had a *p*-value of 0.2 in the bivariable analysis, they were not transferred to a multivariable analysis. The multivariable decomposition regression models found that 85.2% of the decline in unintended pregnancy from 2005 (37%) to 2016 (27%) was attributed to changes in the coefficients (mother’s characteristics) and only 14.8% of the decline was due to changes in the population characteristics (population dynamics) (Table 2).

**Table 2:** Difference due to endowments and coefficients (Ethiopian demographic health survey, 2005 to 2016)

Unintended pregnancy	Coefficient	*p*-value	95% CI	Percent
E = overall changes due to endowments	−0.0160	0.0030	(−0.0265, − 0.0056)	14.8000
C = overall changes due to coefficients	− 0.0923	0.0000	(− 0.1100, − 0. 0743)	85.2000
R = Total Difference	− 0.1083	0.0000	(− 0.1226, − 0. 0940)	

### Non-linear decomposition analysis

The multivariable decomposition comparison showed that the combined effect of all changes in the distribution of population characteristics (endowments) changed the prevalence of unintended pregnancy rate by 1.6% points (a 14.8% decrease = − 0.016033/− 0.10831). The combined effect of all coefficients would have been to decrease unintended pregnancy by 9.3% points (85.2% decrease) due to behavioral effects (Table 2). This finding can be alternatively interpreted as if the composition of the women population composition had remained the same for the 2005 and 2016 surveys, the rate of unintended pregnancy would have increased by 14.8%. Besides, if the effect of the coefficient of each variable had not changed, the rate of unintended pregnancy would have been elevated by 85.2%.

### Difference due to characteristics (Endowment)

Keeping the effect of change in the coefficient of each variable constant, 15% of changes in unintended pregnancy was due to dissimilarity in the composition of women between 2005 and 2016 surveys. Being in the age group of 15–24 years, being a partial decision maker, being a resident of Afar, being a resident of Amhara, being the resident of Oromia region, and being the resident of SNNP all significantly contributed to the change in magnitude of unintended pregnancy. Being a resident of the Somali region and being a partial decision-maker are among the most influential compositional factors.

If women in 2005 had the same behavioral response (i.e., residency in Somalia & partial decision maker) as women in 2016, the prevalence of unintended pregnancy is expected to increase by 14 and 10% percent respectively keeping all other compositional and coefficient factors constant (Table 3).

**Table 3:** Multivariable decomposition result of unintended pregnancy in Ethiopia between 2005 and 2016 Ethiopian demographic health survey

Group 1 = survey year 2005 = 0 (comparison group) Group 2 = survey year 2016 = 1 (reference group)				
Name Of Variable	Difference due to endowments(E)		Difference due to coefficients(C)	
	Coef	Percent shared	Coef.	Percent shared
**Age Group**				
15–24 Years (**reference**)	0	0	0	0
25–34 Years	0.0003	−0.2400	0.0076	−7.0500
15–24 Years	− 0.0001**	0.9100**	0.0084	−7.7600
**Place Of Residency**				
Urban (**reference**)	0	0	0	0
Rural	0.0001	−0.1200	0.032040	− 29.580
**Educational Status**				
None (**reference**)	0	0	0	0
Primary School	0.0021	−1.9700	0.0055	−5.0800
Secondary And Above	−0.0008	0.7000	0.0058*	−5.3400*
**Religion**				
Traditional (**reference**)	0	0	0	0
Orthodox	−0.0068	6.2600	0.0017	−1.5600
Catholic	0.0000	0.0300	−0.0001	0.0600
Protestant	0.0002	−0.2000	− 0.0048	4.4000
Muslim	0.0064	−5.9200	0.0065	−6.0200
**Wealth Status**				
Poor (**reference**)	0	0	0	0
Middle	0.0001	−0.1300	−0.0078	7.2000
Rich	−0.0001	0.0700	−0.0155	14.3300
**Media Exposure**				
Not Exposed (**reference**)	0	0	0	0
Exposed	0.0002	−0.160	− 0.0186*	17.2000*
**Decision Making**				
No (**reference**)	0	0	0	0
Partially	−0.0111**	10.2300**	−0.0471***	43.4600***
Yes	0.0000	−0.0200	−0.0034	3.1700
**Working Status**				
Working (**reference**)	0	0	0	0
Not Working	0.0019	−1.7100	−0.0080*	7.3900*
**Desired Children**				
Above5+ Children (**reference**)	0	0	0	0
Below 5 Children	−0.0006	0.5700	−0.0104	9.6300
**Current Marital Status**				
Married (**reference**)	0	0	0	0
Not Married	−0.0004	0.3200	−0.0005	0.4400
**Perceived Distance To proximate Health Facilities**				
Big Problem (**reference**)	0	0	0	0
Not Big Problem	0.0023	−2.1600	0.0108 *	−10.0100*
**Visit Health Facilities In The Last 12 Months**				
No (**reference**)	0	0	0	0
Yes	−0.0011	1.8300	− 0.0127*	11.7100*
**Knowledge on Family Planning**				
No (**reference**)	0	0	0	0
Yes	0.0257	−23.7300	0.1012*	−93.4700*
**Region Of Residency**				
Tigray (**reference**)	0	0	0	0
Afar	−0.0036*	3.3400*	−0.0039	3.5500
Amhara	−0.0035*	3.2600*	−0.0102	9.4600
Oromia	−0.0052**	4.8500**	−0.0260***	24.0500***
Somali	−0.0154***	14.2100***	−0.0093*	8.6200*
Benishangul	−0.0001	0.0600	−0.0138***	12.7800***
SNNPR	−0.0066**	6.0600**	−0.0174**	16.0800**
Gambela	0.0010	−0.9300	− 0.0065*	6.0000*
Harari	0.0000	−0.0100	−0.0065*	6.0400*
Addis Ababa	0.0006*	−0.5300*	−0.0030	2.8400
Dire Dawa	0.0001	−0.0800	0.00094	−0.8700

One star indicates significance at *p*-value < 0.05, two denote significance at *p*-value< 0.01 and three indicate significance at *p*-value < 0.001

### Difference due to effects of the coefficient

Keeping the effect of change in compositional characteristics stationary, 85% of changes in unintended pregnancy was attributable to the difference in the effect of coefficient (Table 3). This means 85% of the change in unintended pregnancy among reproductive-age women was explained by differences in coefficient across the two surveys. Change of the effect of educational status (secondary and above), autonomy in decision making (partial maker), media exposure (being exposed), perceived distance to nearby health facilities (perceived as a big problem), is not working, being knowledgeable on family planning method, and region of residency (being a resident of Oromia, Somali, Benishangul, SNNPR, Gambela, Harari) were the main explanatory variables for the change of unintended pregnancy over the last 11 years (Table 3).

Knowledge of family planning, being a partial decision-maker, media exposure (exposed), visiting health facilities, and perceived distance to health facilities were factors with the most influential coefficients.

If the influence of the coefficient of knowledge of family planning remained identical between 2005 and 2016, the magnitude of unintended pregnancy would have elevated by 93% in 2016. Similarly, the prevalence of unintended pregnancy would have increased by 43.5%, if the coefficient of partial autonomy in decision making remained the same for the last 11 years. Additionally, the prevalence of unintended pregnancy would have been reduced by 5.3%, if the effect coefficient of being educated to secondary school and above was brought the same in the 2005 and 2016 surveys (Table 3). Therefore, equalizing the differential effects of educational status alone could reduce the risk of unintended pregnancy by 5.3%.

## Discussion

This study identified and evaluated contributions of differences in measurable characteristics and effects of coefficients on change of unintended pregnancy from 2005 to 2016 surveys in Ethiopia. Besides, a considerable reduction in unintended pregnancies was observed between those survey periods (reduced from 37 to 27%). This finding is coherent with other DHS trend analysis carried out in Nigeria, which pointed out that unintended pregnancy reduced from 16.3% in 2003 to 10.6% in 2013 [15]. The increased utilization of modern family planning uptake [28], increased involvement of donor support, elevated public-private partnerships, and the expansion of health extension programs, all of which may have boosted the reduction of unintended pregnancy in Ethiopia [29, 30].

The major portion of change of unintended pregnancy was shared by dissimilarity in the effect of coefficients of explanatory variables across the two surveys. More than 93% of the change in unintended pregnancy was imputable to changes in women who are knowledgeable on modern family planning holding other variables’ coefficient effect constant, a finding in line with other studies from Jamaica [31]. This may be due to women who have good knowledge on modern family planning are more likely to uptake family planning services compared to less those knowledgeable, which prevent unplanned pregnancy [32]. Therefore, we call for health services providers to play a significant role in mothers’ understanding of modern family planning and to approach women based on their knowledge of status.

Change in the coefficient effect of being women partial decision-maker, shifted the number of unintended pregnancies by 43%. This finding is consistent with the study done in Bangladesh [33] and Ghana [34], which declared that as the autonomy scale of women in decision making increases the prevalence of unintended pregnancy decreases. This is because of women having autonomy in health decision especially decision making related to family planning are more likely to utilize modern family planning and in return drives to halt unintended pregnancy [7].

Unequal distribution of the effect of coefficient of distance from health facilities (perceived as not a big problem) contributed positively (i.e., 10% rise) to the prevalence of unintended pregnancy. This finding is supported by studies carried out in Ethiopia [35–37]. This might be justified through, women who reported the distance to proximate health facilities as not big problem are expected to have easy access to family planning and to have good knowledge of other reproductive health-related counseling services [38]. Other studies revealed that as the distance to proximate health facilities increases, the probability of visiting to get services decreases [39–41]. Having a long-distance from facility to residency also decay the probability of visiting facilities through increasing cost of transport and rising issues delaying productive activities [42, 43].

Visit health facilities was one of the reasons for the reduction of unintended pregnancy by 10%.this finding is consistent with the fact that women who visit health facilities have good opportunities to access family planning services to nullify unintended pregnancy [44] as the biggest reason for unintended pregnancies is not using family planning (either not using consistently or appropriately) [45, 46]. This finding has important implications in that equalizing the distribution of women who perceived the distance as not a big problem in 2016 with 2005 would have decreased the prevalence of unintended pregnancy by 10%.

The main strength of this study the authors utilized a large and representative datasets to compare the magnitude of unintended pregnancy between two surveys. Besides, advanced statistical model to address the main contributing factors to the change of unintended pregnancy in Ethiopia. Even though we compared two large datasets to show the change and contributing factors to the change of unintended pregnancy, we were unable to consider other significant contributing variables (cultural, clinical, and other factors) which were not collected by the EDHS program. The fact that the women were asked about the state of their socio-demographic characteristics throughout the previous 5 years prior to the study during the survey period means that this research is not free from recall bias.

Finally, we recommend the upcoming researchers better to do studies that includes the, missed cultural, clinical and other pertinent factors to get good statistical power. As the change in the magnitude of unintended pregnancy in Ethiopia is not satisfactory, deep understanding and knowledge is required through conducting qualitative research methods. For Ethiopian women, we recommend to follow Medias, to know the modern family planning and get awareness about the consequences of unintended pregnancy.

## Conclusion

The present study provide evidence on sources of change in unintended pregnancy between the 2005 and 2016 EDHS. The principal contributing factors to the change of unintended pregnancy were FP knowledge, decision making, media exposure and health facility visits. We conclude that interventional activities better to prioritize women having poor understanding of modern family planning, women having poor participation decision making and women having not satisfactory visits to health facilities.

## Data Availability

The datasets and materials are available at www.dhsprogram.com. Additional data and materials utilized in this study can be obtained from corresponding author for a reasonable request.

## Abbreviations

EDHS: Ethiopian Demographic Health Surveys
EAs: Enumeration areas
FP: Family planning
SNNP: South nations and nationalities people
